# Association between dietary potassium intake and severe headache or migraine in US adults: a population-based analysis

**DOI:** 10.3389/fnut.2023.1255468

**Published:** 2023-09-15

**Authors:** Lisi Xu, Cong Zhang, Yan Liu, Xiuli Shang, Daifa Huang

**Affiliations:** ^1^Department of The Second Cadre Ward, General Hospital of Northern Theater Command, Shen Yang, China; ^2^Department of Neurology, The First Affiliated Hospital of China Medical University, Shen Yang, China

**Keywords:** potassium intake, migraine, hypertension, ATP-sensitive potassium channels, NHANES, restricted cubic spline

## Abstract

**Background:**

Migraine is a prevalent neurovascular headache disorder. The link between dietary potassium and blood pressure has been established. We sought to delineate the relationship between dietary potassium intake and the prevalence of migraines.

**Methods:**

We conducted a cross-sectional analysis using data from the National Health and Nutrition Examination Survey (NHANES) spanning 1999–2004, comprising 10,254 participants aged ≥20 years. Participants who reported severe headaches or migraine in the self-report questionnaire were identified as migraineurs. A 24-h dietary recall methodology was used to assess dietary potassium intake. Multivariate regression analysis and restricted cubic spline (RCS) modeling were utilized to elucidate the relationship between dietary potassium and migraines.

**Results:**

Among the 10,254 participants, 20.1% were identified with migraine or severe headaches. The adjusted odds ratio (OR) for migraine occurrence in the Q2 dietary potassium intake (1771–2,476 mg/d) was 0.84 (95% CI: 0.73–0.97, *p* = 0.021) compared to the lowest quartile (Q1, ≤ 1771 mg/d). The relationship between dietary potassium and migraine exhibited an L-shaped pattern (non-linear, *p* = 0.016) with an inflection at approximately 1439.3 mg/d. In the subgroup analysis, when compared to Q1, who had the lowest dietary potassium intake, the adjusted OR for Q2 in females, those in the medium-high household income group, and with a Body Mass Index (BMI) ≥ 25 kg/m^2^ were as follows: (OR, 0.82; 95% CI, 0.69–0.98), (OR, 0.79; 95% CI, 0.66–0.95), and (OR, 0.78; 95% CI, 0.66–0.93), respectively. No significant interaction was observed across groups after adjusting for all possible covariates.

**Conclusion:**

The relationship between dietary potassium intake and migraine prevalence among US adults appears to follow an L-shaped curve.

## Introduction

Migraine is a debilitating neurological disorder associated with significant pain that affects more than a billion people worldwide, stands as a notable cause of global disability (1, 2), and bears the greatest burden among all diseases (3). According to a global survey, migraine is the second primary contributor to neurological disability (4) and profoundly impacts the quality of life of those affected (5). Migraine is a neurovascular disorder with poor medication tolerability (6). Some research indicates that specific dietary nutrients can exacerbate or reduce migraine attacks (7–9), so other nutrients must be investigated to prevent migraine attacks.

Potassium, pivotal for processes, upholds cellular homeostasis and functionality *via* an array of potassium channels and is instrumental in sensory transmission pathways. Earlier research has illuminated the linkage between migraines and blood pressure, underscoring the antihypertensive effects of dietary potassium. Migraine pathogenesis remains enigmatic. Yet, contemporary studies have unveiled associations with Two-Pore-Domain potassium (K_2P_) channels and ATP-sensitive potassium channels (K_ATP_ channels) (10–13), furnishing novel therapeutic targets to mitigate migraine frequency and intensity. Nonetheless, the nexus between dietary potassium and migraine remains inadequately explored.

Bridging this knowledge gap, we utilized the NHANES database to scrutinize the association between dietary potassium intake and migraines. Further, we aimed to discern if this association varied according to age, gender, BMI, or household income.

## Materials and methods

### Data sources

This cross-sectional investigation harnesses data from the NHANES, orchestrated by the National Center for Health Statistics (NCHS) (14) and the US Centers for Disease Control and Prevention (CDC) (14). Featuring a sophisticated, multi-layered probabilistic design, NHANES assembles nutrition and health-related data from a representative cohort of the civilian, noninstitutionalized US populace. Questionnaires were used to collect relevant information and data on the health status of participants. For our analysis, we procured de-identified data on individuals aged 20 or older who reported severe headaches or migraines, spanning the NHANES 1999–2004 cycle. The NCHS Ethical Review Committee approved the NHANES (15), and participants furnished written informed consent before engagement. Subsequent analyses were exempt from additional Institutional Review Board scrutiny. NHANES data is publicly accessible on the NHANES website.^1^ Adherence to the Strengthening the Reporting of Observational Studies in Epidemiology (STROBE) guidelines ensured rigorous methodological and reporting standards.

### Study design and population

Our analyses were grounded on data amassed from participants across three 2-year NHANES cycles spanning 1999–2004. We implemented the following exclusion criteria to narrow our focus solely on participants with migraines: absence of data regarding dietary potassium intake, lack of demographic data (educational attainment, marital status, smoking habits, household income, physical activity levels, BMI), or absence of medical condition data (hypertension, diabetes mellitus, coronary heart disease, heart failure, angina, heart attack, stroke) and missing data for C-reactive protein (CRP).

### Description of variables

#### Migraine assessment

Severe headaches or migraines were based on self-reports in the miscellaneous pain section of the NHANES questionnaire. Participants affirmatively responding to the question, “During the past 3 months, did you have a severe headache or migraine?” were classified as severe headache sufferers and migraineurs. Given that most individuals with severe headaches or migraines are diagnosed with migraines according to a specific study (16)., it is justified to label respondents reporting severe headaches or migraines in the survey as migraineurs.

#### Assessment of dietary potassium intake

Dietary potassium intake data was extracted from the NHANES nutritional survey utilizing 24-h dietary recalls. The indicated resources for details on specific data collection and calculation methodologies can be reviewed on the website (17, 18). The comprehensive automated recall system meticulously formulates standardized questions and food-specific response options. The Computer-Assisted Dietary Interview System (CADI) was employed to ascertain correct nutritional values based on individual food and beverage consumption, leveraging the Automated Multiple Pass Method (AMPM) from Agriculture (19). An individual’s daily dietary potassium intake was gauged by summing the potassium values from all reported daily consumables. Intake levels were categorized into quartiles with cut-off values of ≤1771 mg/d, 1771–2,476 mg/d, 2,476–3,373 mg/d, and ≥ 3,373 mg/d.

#### Assessment of other variables

Drawing upon existing literature (8, 20–22), critical potential variables include demographic attributes (age, gender, ethnicity, educational attainment, marital status, family income), lifestyle factors (smoking habits, physical activity levels), comorbidities (hypertension, diabetes, stroke, coronary heart disease, heart failure, angina, heart attack), dietary evaluations (intakes of energy, protein, carbohydrates, fats, sodium, magnesium), physical examinations (body mass index), and laboratory investigations (such as CRP). These variables were classified according to insights from prior studies (8, 9, 21, 22). The multi-pass technique was deployed to compute dietary intakes of potassium, sodium, and magnesium based on a 24-h dietary recall (23).

#### Statistical analysis

This research constitutes a secondary analysis of publicly accessible datasets. Sociodemographics, physical activity, health status, and dietary intake were delineated for the entire cohort and varying levels of dietary potassium intake (sorted into quartiles). Continuous variables were represented by means and standard deviations, while categorical variables were expressed *via* counts and frequencies. Continuous variables were analyzed using either the One-way ANOVA or the Kruskal-Wallis test based on their adherence to a normal distribution, whereas categorical variables were examined through ANOVA. The OR and 95% CI concerning the relationship between dietary potassium intake and migraines were evaluated using multivariable logistic regression, benchmarking against the lowest quartile of dietary potassium intake, through model adjustment. Model 1 was adapted to encompass sociodemographic variables like age, gender, and ethnicity. Model 2 expanded upon Model 1 by integrating lifestyle, CRP, and comorbidities. Model 3 included variables related to dietary intake in addition to those adjusted in Model 2.

**Figure 1:**
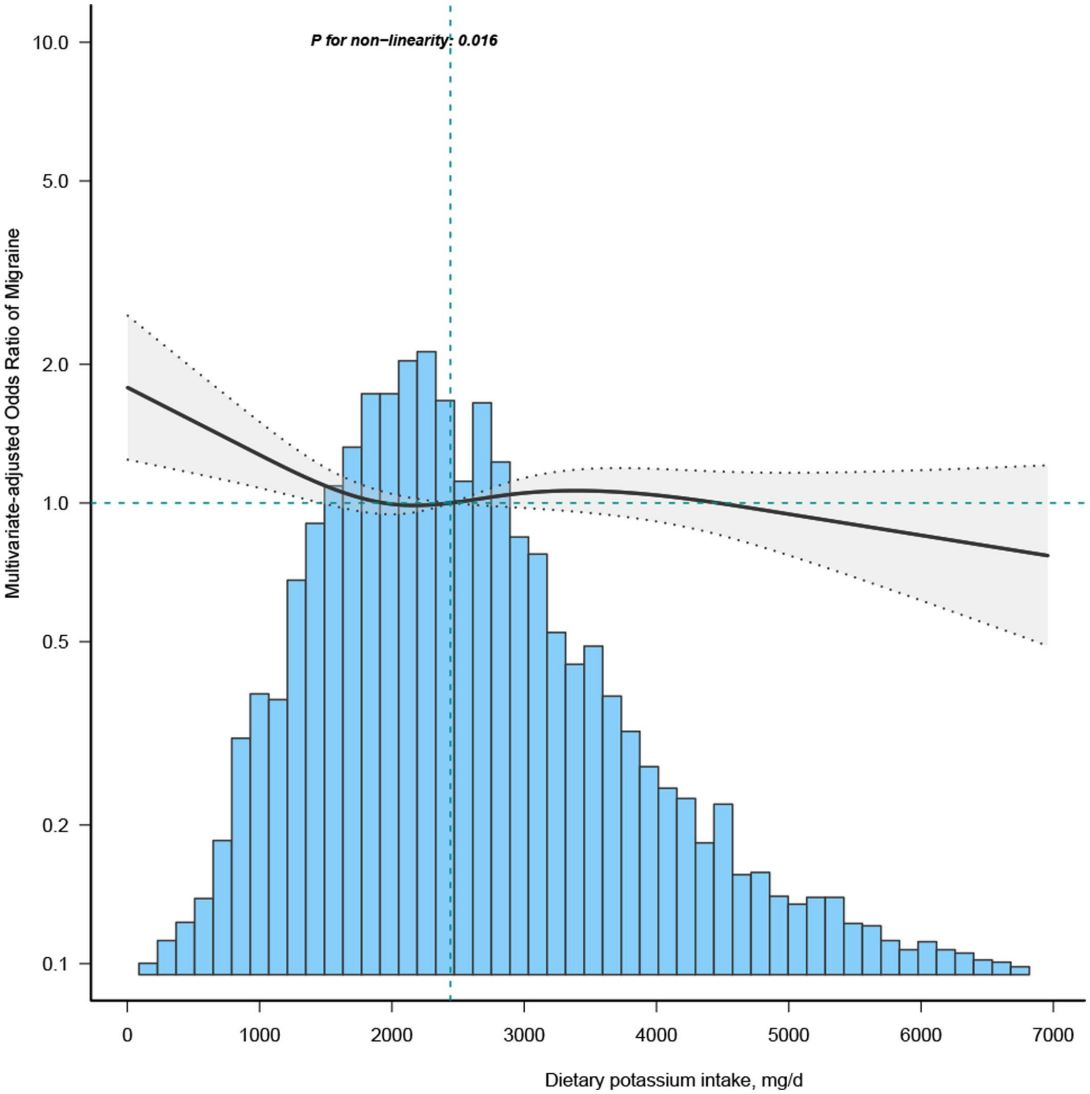
Association between dietary potassium intake and migraine odds ratio. The solid and dashed lines represent the predicted values and 95% confidence intervals, adjusted for all covariates. 99% of the data is shown.

To investigate the non-linear dynamics between dietary potassium intake and migraines, we employed a restricted cubic spline (RCS) and adjusted it according to Model 3 variables (Figure 1). Additionally, stratified analysis was conducted across diverse groups to validate the robustness of the findings.

All statistical analyses were executed using R (version 4.1.1) and Free Statistics software version 1.7. A value of p less than 0.05 was deemed indicative of statistical significance.

## Results

### Study population

**Figure 2:**
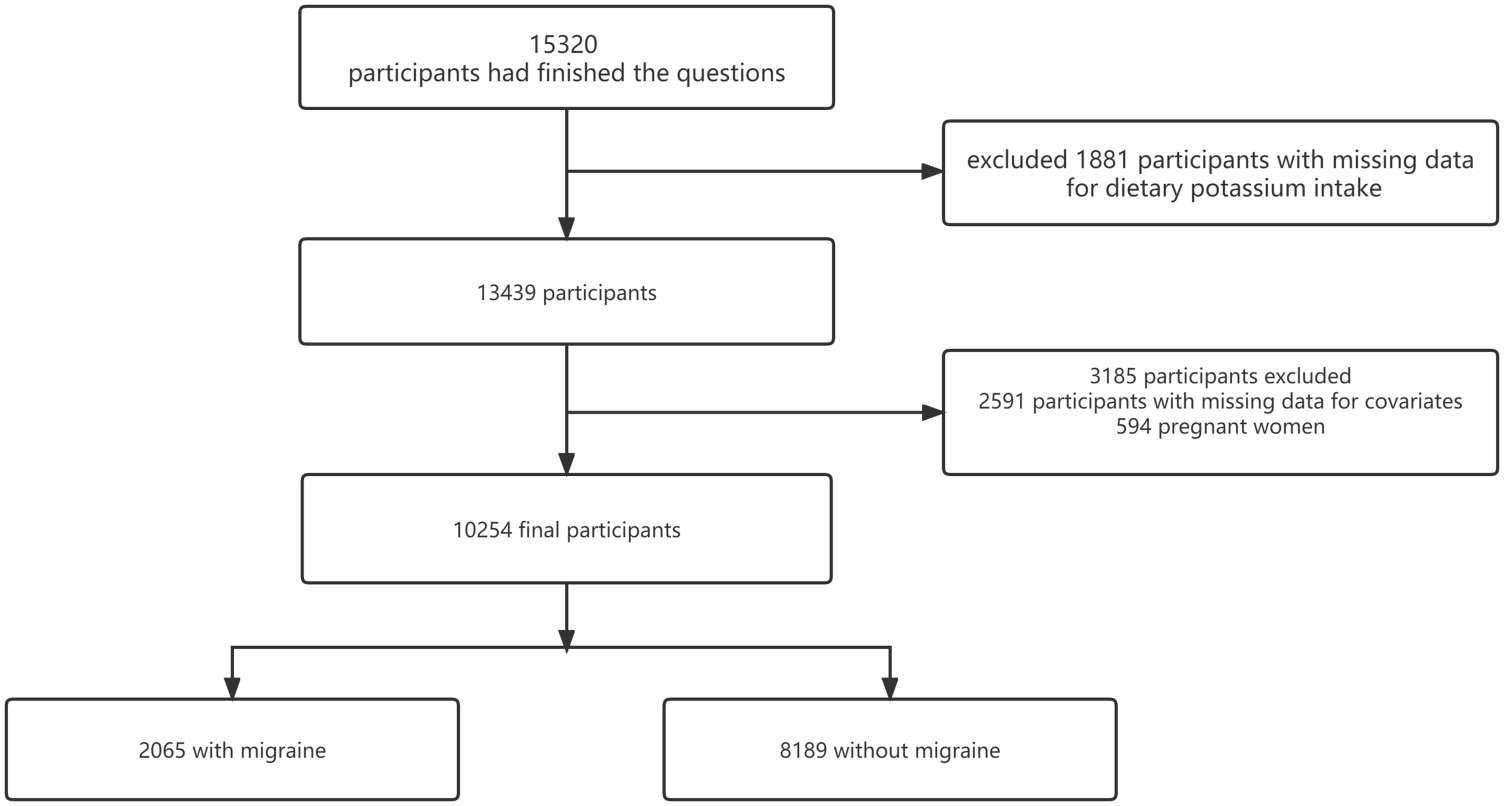
Flowchart of patient enrollment.

From the NHANES 1999–2004 dataset, 15,320 individuals completed the migraine questionnaire. We excluded participants missing dietary potassium data (*n* = 1881), those without key covariate information (*n* = 2,591), and pregnant individuals (*n* = 594). This study yielded a final cohort of 10,254 participants. The inclusion and exclusion process is detailed in Figure 2.

### Characteristics of the participants

We categorize the included participants into four groups based on quartiles of dietary potassium intake and present their baseline characteristics, as shown in Table 1. Among them, 2,065 participants (20.1%) reported experiencing migraines. The cohort’s average age was 50.5 years, with 5,091 (49.6%) females. Individuals with higher potassium intake were observed to be predominantly male, younger, non-smokers, of lighter weight, non-Hispanic white, better educated, with higher income, and consumed greater amounts of energy, protein, carbohydrates, fats, sodium, and magnesium. Most were either married or cohabiting, and a comparatively lower prevalence of comorbidities was noted.

**Table 1:** Population characteristics by categories of dietary potassium intake.

Characteristics	Potassium, mg/d					
	Total	Q1 (≤1771)	Q2 (1771–2,476)	Q3 (2476–3,373)	Q4 (≥3,373)	*p*-value
No	10,254	2,600	2,600	2,543	2,511	
Age, Mean ± SD	50.5 ± 18.5	50.2 ± 19.0	52.5 ± 19.0	51.0 ± 18.0	48.2 ± 17.6	< 0.001
Sex, *n* (%)						< 0.001
Male	5,163 (50.4)	922 (35.5)	1,125 (43.3)	1,365 (53.7)	1751 (69.7)	
Female	5,091 (49.6)	1,678 (64.5)	1,475 (56.7)	1,178 (46.3)	760 (30.3)	
Race/ethnicity, *n* (%)						< 0.001
Non-Hispanic white	5,366 (52.3)	1,093 (42)	1,325 (51)	1,457 (57.3)	1,491 (59.4)	
Non-Hispanic black	1889 (18.4)	735 (28.3)	499 (19.2)	355 (14)	300 (11.9)	
Mexican American	2,232 (21.8)	572 (22)	567 (21.8)	541 (21.3)	552 (22)	
Others	767 (7.5)	200 (7.7)	209 (8)	190 (7.5)	168 (6.7)	
Education level, *n* (%)						< 0.001
<High school	1,491 (14.5)	484 (18.6)	426 (16.4)	306 (12)	275 (11)	
High school	4,102 (40.0)	1,150 (44.2)	1,046 (40.2)	953 (37.5)	953 (38)	
>High school	4,661 (45.5)	966 (37.2)	1,128 (43.4)	1,284 (50.5)	1,283 (51.1)	
Marital status, *n* (%)						< 0.001
Married or living with a partner	6,416 (62.6)	1,430 (55)	1,585 (61)	1706 (67.1)	1,695 (67.5)	
Living alone	3,838 (37.4)	1,170 (45)	1,015 (39)	837 (32.9)	816 (32.5)	
Family income, *n* (%)						< 0.001
Low	2,828 (27.6)	941 (36.2)	738 (28.4)	576 (22.7)	573 (22.8)	
Medium	3,987 (38.9)	1,021 (39.3)	1,027 (39.5)	1,003 (39.4)	936 (37.3)	
High	3,439 (33.5)	638 (24.5)	835 (32.1)	964 (37.9)	1,002 (39.9)	
Smoke status, *n* (%)						< 0.001
Never	5,173 (50.4)	1,336 (51.4)	1,351 (52)	1,289 (50.7)	1,197 (47.7)	
Current	2,296 (22.4)	664 (25.5)	549 (21.1)	519 (20.4)	564 (22.5)	
Former	2,785 (27.2)	600 (23.1)	700 (26.9)	735 (28.9)	750 (29.9)	
Activity, *n* (%)						< 0.001
Sedentary	4,359 (42.5)	1,311 (50.4)	1,191 (45.8)	954 (37.5)	903 (36)	
Moderate	2,908 (28.4)	671 (25.8)	734 (28.2)	796 (31.3)	707 (28.2)	
Vigorous	2,987 (29.1)	618 (23.8)	675 (26)	793 (31.2)	901 (35.9)	
Hypertension, *n* (%)	2,779 (27.1)	771 (29.7)	789 (30.3)	668 (26.3)	551 (21.9)	< 0.001
Diabetes, *n* (%)	1,022 (10.0)	278 (10.7)	286 (11)	244 (9.6)	214 (8.5)	0.013
Stroke, *n* (%)	333 (3.2)	117 (4.5)	97 (3.7)	66 (2.6)	53 (2.1)	< 0.001
Coronary heart disease, *n* (%)	487 (4.7)	121 (4.7)	131 (5)	130 (5.1)	105 (4.2)	0.382
Heart failure, *n* (%)	320 (3.1)	111 (4.3)	86 (3.3)	67 (2.6)	56 (2.2)	< 0.001
Angina, *n* (%)	409 (4.0)	115 (4.4)	102 (3.9)	101 (4)	91 (3.6)	0.535
Heart attack, *n* (%)	468 (4.6)	131 (5)	126 (4.8)	116 (4.6)	95 (3.8)	0.149
Migraine, *n* (%)	2065 (20.1)	634 (24.4)	499 (19.2)	483 (19)	449 (17.9)	< 0.001
Body mass index, Mean ± SD	28.4 ± 6.2	28.8 ± 6.6	28.3 ± 6.0	28.4 ± 6.2	27.9 ± 5.9	< 0.001
Energy, Mean ± SD	2120.1 ± 1028.5	1341.2 ± 572.6	1842.6 ± 644.1	2254.5 ± 737.9	3077.6 ± 1164.8	< 0.001
Protein intake, Mean ± SD	79.6 ± 42.0	47.1 ± 21.3	67.5 ± 23.8	85.3 ± 29.1	120.2 ± 48.5	< 0.001
Carbohydrate intake, Mean ± SD	262.2 ± 134.6	172.0 ± 87.1	228.9 ± 94.6	276.4 ± 105.3	375.7 ± 151.6	< 0.001
Fat, Mean ± SD	78.9 ± 46.2	48.3 ± 25.5	69.0 ± 30.7	85.4 ± 37.3	114.5 ± 57.3	< 0.001
sodium, Mean ± SD	3288.1 ± 1823.3	2052.4 ± 1069.0	2873.6 ± 1215.7	3523.6 ± 1454.6	4758.2 ± 2178.4	< 0.001
Magnesium, Mean ± SD	274.3 ± 144.8	142.6 ± 56.6	222.1 ± 60.0	296.0 ± 75.4	442.9 ± 151.9	< 0.001
C-Reactive Protein, Median (IQR)	0.2 (0.1, 0.5)	0.3 (0.1, 0.6)	0.2 (0.1, 0.5)	0.2 (0.1, 0.5)	0.2 (0.1, 0.4)	< 0.001

Categorical variables were represented by proportions (%) while continuous variables were described by the mean (standard deviation, SD) or median (interquartile range, IQR), as appropriate.

### Association between dietary potassium intake and migraine

Most of the covariates in the univariate analysis demonstrated an association with migraine (Supplementary Table S1). Subsequent multivariate logistic regression analyses were performed, adjusting for all covariates to discern the relationship between potassium (segmented by quartiles) and migraine (Table 2). Upon controlling for potential confounders in the multivariate regression analysis model 3, the OR for the Q2 group (1771–2,476 mg/day) stood at 0.84 (95% CI: 0.73–0.97) compared to the Q1 group (≤ 1771 mg/day). The RCS depicted an L-shaped curve for the relationship between dietary potassium and migraine, suggesting non-linearity (*p* = 0.016) (Figure 1).

**Table 2:** Association between dietary potassium intake and migraine.

Variable	n. total	crude. OR 95CI	Crude. *p*_value	Model 1		Model 2		Model 3	
Dietary Potassium									
(mg/day)									
Q1 (≤1771)	2,600	1 (Reference)		1 (Reference)		1 (Reference)		1 (Reference)	
Q2 (1771–2,476)	2,600	0.74 (0.65 ~ 0.84)	<0.001	0.82 (0.71 ~ 0.94)	0.004	0.86 (0.75 ~ 0.99)	0.04	0.84 (0.73 ~ 0.97)	0.021
Q3 (2476–3,373)	2,543	0.73 (0.64 ~ 0.83)	<0.001	0.86 (0.75 ~ 0.99)	0.031	0.95 (0.82 ~ 1.1)	0.484	0.9 (0.77 ~ 1.06)	0.205
Q4 (≥3,373)	2,511	0.68 (0.59 ~ 0.77)	<0.001	0.85 (0.74 ~ 0.99)	0.033	0.95 (0.82 ~ 1.11)	0.535	0.87 (0.71 ~ 1.06)	0.169
Trend.test			<0.001		0.049		0.748		0.241

Q, quartiles; OR, odds ratio; CI, confidence interval. Model 1 was adjusted for age, sex, race/ethnicity. Model 2 was adjusted for sociodemographics (age, sex, race/ethnicity, education level, marital status, family income), smoking status, physical activity, hypertension, diabetes, stroke, coronary heart disease, heart failure, angina, heart attack, body mass index, and C-reactive protein. Model 3 was adjusted for sociodemographic (age, sex, race/ethnicity, education level, marital status, family income), smoking status, physical activity, hypertension, diabetes, stroke, coronary heart disease, heart failure, angina, heart attack, body mass index, C-reactive protein, energy consumption, protein consumption, carbohydrate consumption, fat consumption, sodium consumption.

In the threshold evaluation, the inflection point value for dietary potassium was determined at 1439.3 mg/day (dietary potassium as a continuous variable, with OR value calculated per 100 mg/d decrease) with an OR of 0.952 (95% CI: 0.906–1) (Table 3). This result inferred that with every 0.1 g increment in daily potassium intake, migraine occurrence was reduced by 4.8%. Beyond the 1439.3 mg/day mark, dietary potassium’s association with migraine vanished, indicating that escalating potassium intake did not further diminish migraine prevalence (Table 3). The discrepancy between the dietary potassium threshold and the recommended intake is presented in Supplementary Table S2.

**Table 3:** Threshold effect analysis of the relationship of potassium intake with migraine.

Potassium intake 100 mg/day	Adjusted model	
	OR (95%CI)	*p*-value
<14.39	0.952 (0.906 ~ 1)	0.049
≥14.39	0.998 (0.991–1.006)	0.632
Log-likelihood ratio test		0.039

OR, odds ratio; CI, confidence interval. Adjusted for sociodemographic (age, sex, race/ethnicity, education level, marital status, family income), smoking status, physical activity, hypertension, diabetes, stroke, coronary heart disease, heart failure, angina, heart attack, body mass index, C-reactive protein, energy consumption, protein consumption, carbohydrate consumption, fat consumption, sodium consumption. Only 99% of the data is displayed.

### Subgroup analyses

Subgroup evaluations were conducted to gauge the effect of specific variables (like gender, age, family earnings, and BMI) on the relationship between dietary potassium and migraine (Table 4). No associations were discerned across the subgroups, and no substantial interaction effects emerged among them.

**Table 4:** Subgroup analysis by dietary potassium intake.

Subgroup	Event (%)	OR (95%CI)	Subgroup	Event (%)	OR (95%CI)	P for interaction
Sex						0.499
Male			Female			
Q1 (≤1771)	134 (14.5)	1(reference)	Q1 (≤1771)	500 (29.8)	1 (reference)	
Q2 (1771–2,476)	145 (12.9)	0.98 (0.75 ~ 1.28)	Q2 (1771–2,476)	354 (24)	0.82 (0.69 ~ 0.98)	
Q3 (2476–3,373)	193 (14.1)	1.09 (0.83 ~ 1.43)	Q3 (2476–3,373)	290 (24.6)	0.86 (0.7 ~ 1.06)	
Q4 (≥3,373)	257 (14.7)	1.02 (0.75 ~ 1.39)	Q4 (≥3,373)	192 (25.3)	0.85 (0.65 ~ 1.12)	
Age, y						0.715
20–50			>50			
Q1 (≤1771)	414 (31.3)	1 (reference)	Q1 (≤1771)	220 (17.2)	1 (reference)	
Q2 (1771–2,476)	319 (26.3)	0.85 (0.7 ~ 1.03)	Q2 (1771–2,476)	180 (13)	0.84 (0.67 ~ 1.07)	
Q3 (2476–3,373)	308 (24.3)	0.82 (0.67 ~ 1.01)	Q3 (2476–3,373)	175 (13.7)	0.98 (0.75 ~ 1.28)	
Q4 (≥3,373)	315 (22.4)	0.82 (0.64 ~ 1.05)	Q4 (≥3,373)	134 (12.1)	0.88 (0.62 ~ 1.23)	
Family income						0.262
Low			Medium or high			
Q1 (≤1771)	253 (26.9)	1 (reference)	Q1 (≤1771)	381 (23)	1 (reference)	
Q2 (1771–2,476)	183 (24.8)	0.95 (0.74 ~ 1.2)	Q2 (1771–2,476)	316 (17)	0.79 (0.66 ~ 0.95)	
Q3 (2476–3,373)	140 (24.3)	0.95 (0.72 ~ 1.25)	Q3 (2476–3,373)	343 (17.4)	0.88 (0.72 ~ 1.06)	
Q4 (≥3,373)	149 (26)	1.05 (0.74 ~ 1.47)	Q4 (≥3,373)	300 (15.5)	0.8 (0.63 ~ 1.02)	
BMI <25			BMI ≥25			0.266
Q1 (≤1771)	182 (23.1)	1 (reference)	Q1 (≤1771)	452 (24.9)	1 (reference)	
Q2 (1771–2,476)	166 (20.6)	0.97 (0.75 ~ 1.26)	Q2(1771–2,476)	333 (18.5)	0.78 (0.66 ~ 0.93)	
Q3 (2476–3,373)	145 (18)	0.92 (0.69 ~ 1.22)	Q3 (2476–3,373)	338 (19.4)	0.89 (0.73 ~ 1.08)	
Q4 (≥3,373)	143 (17.1)	0.88 (0.62 ~ 1.25)	Q4 (≥3,373)	306 (18.3)	0.86 (0.68 ~ 1.1)	

Except for the stratification component itself, each stratification factor was adjusted for all other variables (age, sex, race/ethnicity, education level, marital status, family income, smoking status, physical activity, hypertension, diabetes, stroke, coronary heart disease, heart failure, angina, heart attack, body mass index, C-reactive protein, energy consumption, protein consumption, carbohydrate consumption, fat consumption, sodium consumption).

## Discussion

This cross-sectional study unveiled an L-shaped correlation between potassium intake and migraine, pinpointing a transition threshold at approximately 1439.3 mg/day. Compared to the participants in group Q1, those in group Q4 in the unadjusted model, as well as those in Q4 of model 1 (after adjusting for age, ethnicity, and gender), had a lower risk of migraine prevalence, with odds ratios (OR) of 0.68 (95% CI: 0.59–0.77) and 0.85 (95% CI: 0.74–0.99), respectively. Those in the Q2 group, with elevated potassium consumption, showed diminished migraine risks (OR:0.84; 95%CI: 0.73–0.97) compared to Q1, after adjusting for demographic data, lifestyle habits, comorbidities, and nutritional intake. After adjusting for pertinent confounders and executing an inflection point analysis, it was determined that when the dietary potassium intake was less than 1439.3 mg/day, the prevalence of migraines diminished progressively with increasing potassium intake. However, once dietary potassium intake exceeded or equaled 1439.3 mg/day, the prevalence of migraines remained stable despite further increases in potassium intake. Both the subgroup and sensitivity analyses, adjusted for confounders, affirmed this observed relationship. To our understanding, this is the pioneering cross-sectional study within the U.S. adult demographic probing the connection between dietary potassium and migraine.

Dietary micronutrients are pivotal, underpinning numerous metabolic processes. Potassium is crucial for sustaining regular human metabolism. It is the predominant cation within cellular structures, vital for maintaining the standard form and function of cells. However, this study found that many American adults consume less potassium than the recommended daily intake, a deficiency particularly noticeable in women (Table 1), consistent with previous studies (24, 25). This discrepancy can be attributed to Western dietary habits. Predominantly found in fruits and vegetables and only in trace amounts in grains, potassium intake in Western diets is often compromised due to a higher grain consumption at the expense of fruits and vegetables. Elevated potassium intake has proven beneficial for reducing blood pressure (26), and its increased consumption has been inversely related to numerous chronic diseases. Existing research suggests a dietary potassium intake exceeding 3,500 mg/day as a proactive measure against hypertension. Dietary guidelines set by esteemed organizations such as the American Heart Association (AHA), the Institute of Medicine (IOM), the US Department of Health and Human Services, and the Department of Agriculture (USDA) unanimously advocate for potassium-rich food choices to mitigate adverse health outcomes (19, 27).

However, in this study, 7,996 individuals (78%) registered potassium intake levels below 3,500 mg/day, including 3,565 (44.6%) males and 4,431 (55.4%) females, a distinction statistically significant difference between the genders (*p* < 0.001) (Supplementary Table S2). Of the surveyed participants, 1,569 individuals (or 15.3% of the sample, with women comprising 65.8%) reported a potassium intake below the identified inflection point of 1439.3 mg/day. This result underscores that many Americans, especially women, might overlook the importance of dietary potassium and not meet the suggested daily intake. This oversight might contribute to the elevated prevalence of migraines in women compared to men. As illustrated in Table 1, daily dietary potassium intake is relatively lower among specific demographic groups, including women, older individuals, non-Hispanic blacks, those with limited education, individuals living alone, those with lower income, smokers, sedentary individuals, those with historical co-morbidities, and those with lower intake of energy, protein, fat, carbohydrate, and magnesium. Comprehensive multifactorial regression analyses were employed to mitigate potential confounder effects on the outcomes. Moreover, we conducted subgroup analyses supporting a consistent relationship between dietary potassium and migraines. Although these factors were considered, the potential influence of unobserved confounders cannot be entirely dismissed. In clinical settings, it’s essential to bolster healthcare awareness among these groups, emphasizing the significance of balanced nutrition and encouraging the inclusion of potassium-rich foods in daily diets. Such measures might reduce the national migraine prevalence, but more research is needed to confirm this.

The present study suggests that potassium may influence migraine. A study from the 1950s observed that sufferers excreted elevated levels of sodium and potassium in their urine, hinting at a potential connection between these electrolytes and migraine pathophysiology (28). Subsequent research has primarily focused on the relationship between sodium and migraine (24, 25, 29, 30). However, the role of potassium has largely remained unexplored, signifying a clear need for more detailed studies examining the correlation between dietary potassium and migraines.

A study assessing the correlation between 24-h urinary sodium and potassium intake with migraines within an Iranian sample discovered that elevated 24-h urinary sodium levels corresponded with migraines. However, the association between potassium intake and migraines was not statistically significant (25). In contrast, the present cross-sectional study identified a negative association between dietary potassium intake and the prevalence of migraines within the general U.S. population. This inverse relationship persisted even after adjustments for factors like ethnicity and gender. Additionally, when accounting for other potential confounders, such as education and medical history, the negative correlation between dietary potassium and migraine remained evident, albeit to a certain degree. Further stratified and sensitivity analyses reinforced this negative association, highlighting the stability of our findings, even after adjusting for relevant confounders, which offers a fresh perspective, supplementing insights from previous studies. To date, the relationship between dietary potassium and migraines remains under-investigated. Established research confirms that augmented dietary potassium intake can reduce blood pressure (26), and there’s a consistent association between migraines and blood pressure. Our results imply that an enhanced dietary potassium intake, within certain parameters, might mitigate migraines. This result could be attributed to its potential role in reducing blood pressure, curbing free radical formation, and inhibiting vascular smooth muscle proliferation. The pathophysiological mechanisms of migraine remain elusive. Numerous studies propose migraines emerge when oxidative stress levels surpass the body’s antioxidant capability to counteract such stress (31). Evidence suggests that dietary potassium’s protective influence on endothelial cells potentially prevents vascular damage from oxidative stress (32, 33). Migraines are predominantly perceived as neurovascular sensory threshold disorders (34). Various chemicals identified in human trials can precipitate migraines, with cephalic artery dilation (35–39) *via* the subsequent activation of vascular smooth muscle ATP-sensitive potassium (K_ATP_) channels (40) being the most prevalent. The essential function of the ATPase enzyme lies in ensuring homeostasis and facilitating the intracellular movement of extracellular potassium (41). K_ATP_ channels are implicated in migraines (12), with the most compelling evidence being that the K_ATP_ channel opener, levcromakalim, can induce migraines, as demonstrated by Al-Karagholi et al. (10, 11). The body’s pain signal perception is intertwined with ion channel activity. Within this spectrum, the K_2P_ channels are paramount and are viewed as a potential therapeutic target for migraine treatments (42–44). Moreover, imbalances in extracellular potassium homeostasis can instigate migraines with aura (45). Elevated extracellular potassium concentrations are associated with various disorders (46–48), including migraine (49). In general, cations are indispensable for brain functionality and have been pinpointed in the pathophysiology of various disorders, including migraines (50). Research has been done to assess the connection between 24-h urinary sodium and urinary potassium and migraine in an Iranian population (25). These results suggest that increasing dietary potassium may be critical in lowering migraine frequency.

## Limitations

Several limitations of this study should be noted. First, the cross-sectional design of this research prohibits the establishment of a causal relationship between dietary potassium intake and migraine. Future studies employing a cohort design will be crucial for purpose. Second, our data was sourced from the NHANES database, which provides migraine-related information solely for the 1999–2004 cycle. Hence, we could not validate our findings across other cycles. Third, the participants comprised exclusively U.S. adults. Therefore, generalizing these findings to broader demographics might be challenging, given that factors like ethnicity, dietary habits, and educational background significantly influence the nexus between dietary potassium and migraines. Fourth, while we incorporated salient covariates from previous studies (8, 9, 21, 22, 51, 52), it’s essential to recognize that daily potassium intake is contingent on myriad factors. Despite considering elements previously associated with migraines, potential unmeasured confounders that were not addressed in our analysis might still exist. Fifth, potential recall bias is another limitation since dietary data stemmed from a 24-h self-reported dietary review. However, the NHANES dataset is reputed for its comprehensive and high-quality dietary information. Finally, the results of this study rely on self-reported data and, as such, aren’t definitively classifiable. Nevertheless, according to the American Migraine Prevalence and Prevention Study, 17.4% of respondents reported experiencing severe headaches. Within this subgroup, 11.8% satisfied the criteria for migraine diagnosis as per the International Classification of Headache Disorders (ICHD) (16), with an additional 4.6% meeting the criteria for probable migraines. Given the demographic resemblance of our study’s participants to population samples from epidemiological surveys, it’s plausible to deduce that most of those reporting migraines or severe headaches indeed suffer from migraines. Given these limitations, our subsequent research endeavors will employ more stringent headache classification criteria, categorize patients, and initiate prospective investigations to more accurately determine which type of migraine patients might benefit from augmented dietary potassium intake.

## Conclusion

This research represents a pioneering effort to elucidate the L-shaped correlation between dietary potassium intake and migraines, highlighting an inflection point at 1439.3 mg/day. The findings underscore the potential prophylactic benefits of increasing dietary potassium to thwart migraines.

## Author’s note

LX is a current medical doctor at The First Affiliated Hospital of China Medical University, where her main research interests are in cerebrovascular diseases and headache disorders. And she works as an attending physician at the Second Cadre Ward of the General Hospital of Northern Theater Command.

CZ is an attending physician at the Second Cadre Ward of the General Hospital of Northern Theater Command, who focuses on geriatric diseases, cerebrovascular diseases and headaches.

YL is an associate chief physician at the Second Cadre Ward of the General Hospital of Northern Theater Command, who specializes in geriatric diseases and headaches.

XS is a professor of the Department of Neurology, West China Hospital of Sichuan University, focusing on studies of cerebrovascular diseases，headaches and cognition.

DH is a chief doctor in the General Hospital of Northern Theater Command, and his work focuses on geriatric diseases, cerebrovascular diseases and headaches.

## Data availability statement

The original contributions presented in the study are included in the article/Supplementary material, further inquiries can be directed to the corresponding authors.

## Ethics statement

The studies involving humans were approved by the National Center for Health Statistics (NCHS). The studies were conducted in accordance with the local legislation and institutional requirements. Written informed consent for participation was not required from the participants or the participants’ legal guardians/next of kin in accordance with the national legislation and institutional requirements. Written informed consent was obtained from the individual(s) for the publication of any potentially identifiable images or data included in this article.

## Author contributions

LX: Writing – original draft, Writing – review & editing, Conceptualization, Data curation, Resources, Validation, Visualization. CZ: Writing – review & editing. YL: Writing – review & editing. XS: Supervision, Writing – review & editing. DH: Writing – review & editing.

## Funding

The author(s) declare that no financial support was received for the research, authorship, and/or publication of this article.

## Conflict of interest

The authors declare that the research was conducted in the absence of any commercial or financial relationships that could be construed as a potential conflict of interest.

## Publisher’s note

All claims expressed in this article are solely those of the authors and do not necessarily represent those of their affiliated organizations, or those of the publisher, the editors and the reviewers. Any product that may be evaluated in this article, or claim that may be made by its manufacturer, is not guaranteed or endorsed by the publisher.

## Figures and Tables

**Table:** 

AHA	the American Heart Association
AMPM	Agriculture used the Automated Multiple Pass Method
BMI	Body mass index
CDI	Computer-Assisted Dietary Interview System
CI	confidence interval
CRP	C-reactive protein
ICHD	International Classification of Headache Disorders
IOM	the Institute of Medicine
K_ATP_ channels	ATP-sensitive potassium channels
K_2P_ channels	Two-Pore-Domain potassium channels
NHANES	National Health and Nutrition Examination Survey
NCHS	National Center for Health Statistics
OR	odds ratio
Q	quartile
RCS	restricted cubic spline
STROBE	Strengthening the Reporting of Observational Studies in Epidemiology
USDA	the US Department of Health and Human Services and the Department of Agriculture

## Footnotes

^1^ https://www.cdc.gov/nchs/nhanes/index.htm

## References

[1] Collaborators GBDH. Global, regional, and national burden of migraine and tension-type headache, 1990-2016: a systematic analysis for the global burden of Disease study 2016. Lancet Neurol. (2018) 17:954–76. doi: 10.1016/S1474-4422(18)30322-3 30353868 PMC6191530

[2] Steiner TJ Stovner LJ Jensen R Uluduz D Katsarava Z. Lifting the burden: the global campaign against H. migraine remains second among the world's causes of disability, and first among young women: findings from GBD2019. J Headache Pain. (2020) 21:137. doi: 10.1186/s10194-020-01208-0 33267788 PMC7708887

[3] Disease and Injury Incidence and Prevalence Collaborators. Global, regional, and national incidence, prevalence, and years lived with disability for 354 diseases and injuries for 195 countries and territories, 1990-2017: a systematic analysis for the global burden of Disease study 2017. Lancet. (2018) 392:1789–858. doi: 10.1016/S0140-6736(18)32279-7 30496104 PMC6227754

[4] Feigin VL Nichols E Alam T Bannick MS Beghi E Blake N. Global, regional, and national burden of neurological disorders, 1990–2016: a systematic analysis for the global burden of Disease study 2016. Lancet Neurol. (2019) 18:459–80. doi: 10.1016/S1474-4422(18)30499-X, PMID: 30879893 PMC6459001

[5] Silberstein SD. Migraine. Lancet. (2004) 363:381–91. doi: 10.1016/S0140-6736(04)15440-8 15070571

[6] Hepp Z Dodick DW Varon SF Chia J Matthew N Gillard P. Persistence and switching patterns of oral migraine prophylactic medications among patients with chronic migraine: a retrospective claims analysis. Cephalalgia. (2017) 37:470–85. doi: 10.1177/0333102416678382, PMID: 27837173 PMC5405847

[7] Gazerani P. Migraine and diet. Nutrients. (2020) 12:6. doi: 10.3390/nu12061658 PMC7352457 32503158

[8] Meng SH Wang MX Kang LX Fu JM Zhou HB Li X. Dietary intake of calcium and magnesium in relation to severe headache or migraine. Front Nutr. (2021) 8:653765. doi: 10.3389/fnut.2021.653765, PMID: 33748178 PMC7973018

[9] Meng SH Zhou HB Li X Wang MX Kang LX Fu JM. Association between dietary iron intake and serum ferritin and severe headache or migraine. Front Nutr. (2021) 8:685564. doi: 10.3389/fnut.2021.685564, PMID: 34295917 PMC8289886

[10] Al-Karagholi MA Ghanizada H Nielsen CAW Hougaard A Ashina M. Opening of ATP sensitive potassium channels causes migraine attacks with aura. Brain. (2021) 144:2322–32. doi: 10.1093/brain/awab136, PMID: 33768245

[11] Al-Karagholi MA Hansen JM Guo S Olesen J Ashina M. Opening of ATP-sensitive potassium channels causes migraine attacks: a new target for the treatment of migraine. Brain. (2019) 142:2644–54. doi: 10.1093/brain/awz199, PMID: 31292608

[12] Christensen SL Rasmussen RH Cour S Ernstsen C Hansen TF Kogelman LJ. Smooth muscle ATP-sensitive potassium channels mediate migraine-relevant hypersensitivity in mouse models. Cephalalgia. (2022) 42:93–107. doi: 10.1177/03331024211053570, PMID: 34816764

[13] Clement A Guo S Jansen-Olesen I Christensen SL. ATP-sensitive potassium channels in migraine: translational findings and therapeutic potential. Cells. (2022) 11:2406. doi: 10.3390/cells11152406, PMID: 35954249 PMC9367966

[14] Cabral C Silveira M. Classification of Alzheimer's disease from FDG-PET images using favourite class ensembles. Annu Int Conf IEEE Eng Med Biol Soc. (2013) 2013:2477–80. doi: 10.1109/EMBC.2013.6610042, PMID: 24110229

[15] Zeng HM Han HB Zhang QF Bai H. Application of modern neuroimaging technology in the diagnosis and study of Alzheimer's disease. Neural Regen Res. (2021) 16:73–9. doi: 10.4103/1673-5374.286957, PMID: 32788450 PMC7818875

[16] Buse DC Loder EW Gorman JA Stewart WF Reed ML Fanning KM. Sex differences in the prevalence, symptoms, and associated features of migraine, probable migraine and other severe headache: results of the American migraine Prevalence and prevention (AMPP) study. Headache. (2013) 53:1278–99. doi: 10.1111/head.12150, PMID: 23808666

[17] RR B. Assessment of the US diet in national nutrition surveys: national collaborative efforts and NHANES. Am J Clin Nutr. (1994) 59:164S–7S. doi: 10.1093/ajcn/59.1.164S, PMID: 8279416

[18] Yee E Popuri K Beg MF. Quantifying brain metabolism from FDG-PET images into a probability of Alzheimer's dementia score. Hum Brain Mapp. (2020) 41:5–16. doi: 10.1002/hbm.24783, PMID: 31507022 PMC7268066

[19] Food Surveys Research Group: USDA ARS;Agriculture Research Service FSRG. Food and nutrient database for dietary studies, 5.0. (2023) (Accessed January 20, 2023). Available at: https://www.ars.usda.gov/northeast-area/beltsville-md-bhnrc/beltsville-human-nutrition-research-center/food-surveys-research-group/

[20] Li H Krall JR Frankenfeld C Slavin M. Nutritional intake of riboflavin (vitamin B2) and migraine: a cross-sectional analysis of the National Health and nutrition examination survey (NHANES) 2001-2004. Nutr Neurosci. (2022) 1-10:1–10. doi: 10.1080/1028415X.2022.2126760 36175363

[21] Liu H Wang L Chen C Dong Z Yu S. Association between dietary niacin intake and migraine among American adults: National Health and nutrition examination survey. Nutrients. (2022) 14:3052. doi: 10.3390/nu14153052 35893904 PMC9330821

[22] Liu H Wang Q Dong Z Yu S. Dietary zinc intake and migraine in adults: a cross-sectional analysis of the National Health and nutrition examination survey 1999-2004. Headache. (2023) 63:127–35. doi: 10.1111/head.14431, PMID: 36588459

[23] Moshfegh AJRD Baer DJ Murayi T Clemens JC Rumpler WV Paul DR. The US Department of Agriculture Automated Multiple-Pass Method reduces bias in the collection of energy intakes. Am J Clin Nutr. (2008) 88:324–32. doi: 10.1093/ajcn/88.2.324, PMID: 18689367

[24] Amer M Woodward M Appel LJ. Effects of dietary sodium and the DASH diet on the occurrence of headaches: results from randomised multicentre DASH-sodium clinical trial. BMJ Open. (2014) 4:e006671. doi: 10.1136/bmjopen-2014-006671, PMID: 25500372 PMC4265150

[25] Arab A Khorvash F Heidari Z Askari G. Is there a relationship between dietary sodium and potassium intake and clinical findings of a migraine headache? Br J Nutr. (2022) 127:1839–48. doi: 10.1017/S000711452100283X, PMID: 34378504

[26] Chobanian AV Bakris GL Black HR Cushman WC Green LA Izzo JL Jr. Seventh report of the joint National Committee on prevention, detection, evaluation, and treatment of high blood pressure. Hypertension. (2003) 42:1206–52. doi: 10.1161/01.HYP.0000107251.49515.c2 14656957

[27] Institute of Medicine. Dietary reference intakes for water, potassium, sodium, chloride, and sulfate: Washington. DC: The National Academies Press (2005).

[28] WW S. Renal excretion of fluid and electrolytes in association with vascular headache. Psychosom Med. (1956) 18:252–8. doi: 10.1097/00006842-195605000-00006 13323182

[29] Chen L Zhang Z Chen W Whelton PK Appel LJ. Lower sodium intake and risk of headaches: results from the trial of nonpharmacologic interventions in the elderly. Am J Public Health. (2016) 106:1270–5. doi: 10.2105/AJPH.2016.303143, PMID: 27077348 PMC4902761

[30] Pogoda JM Gross NB Arakaki X Fonteh AN Cowan RP Harrington MG. Severe headache or migraine history is inversely correlated with dietary sodium intake: NHANES 1999-2004. Headache. (2016) 56:688–98. doi: 10.1111/head.12792, PMID: 27016121 PMC4836999

[31] Gross EC Lisicki M Fischer D Sandor PS Schoenen J. The metabolic face of migraine - from pathophysiology to treatment. Nat Rev Neurol. (2019) 15:627–43. doi: 10.1038/s41582-019-0255-4, PMID: 31586135

[32] Kido M Ando K Onozato ML Tojo A Yoshikawa M Ogita T. Protective effect of dietary potassium against vascular injury in salt-sensitive hypertension. Hypertension. (2008) 51:225–31. doi: 10.1161/HYPERTENSIONAHA.107.098251, PMID: 18158352

[33] Smiljanec K Mbakwe A Ramos Gonzalez M Farquhar WB Lennon SL. Dietary potassium attenuates the effects of dietary sodium on vascular function in salt-resistant adults. Nutrients. (2020) 12:1206. doi: 10.3390/nu12051206, PMID: 32344796 PMC7281996

[34] Peng KP May A. Migraine understood as a sensory threshold disease. Pain. (2019) 160:1494–501. doi: 10.1097/j.pain.0000000000001531, PMID: 31219950

[35] Deen M Correnti E Kamm K Kelderman T Papetti L Rubio-Beltran E. Blocking CGRP in migraine patients - a review of pros and cons. J Headache Pain. (2017) 18:96. doi: 10.1186/s10194-017-0807-1, PMID: 28948500 PMC5612904

[36] Kruuse C Thomsen LL Birk S Olesen J. Migraine can be induced by sildenafil without changes in middle cerebral artery diameter. Brain. (2003) 126:241–7. doi: 10.1093/brain/awg009, PMID: 12477710

[37] Lassen LH Haderslev PA Jacobsen VB Iversen HK Sperling B Olesen J. CGRP May play a causative role in migraine. Cephalalgia. (2002) 22:54–61. doi: 10.1046/j.1468-2982.2002.00310.x 11993614

[38] Pellesi L Al-Karagholi MA Chaudhry BA Lopez CL Snellman J Hannibal J. Two-hour infusion of vasoactive intestinal polypeptide induces delayed headache and extracranial vasodilation in healthy volunteers. Cephalalgia. (2020) 40:1212–23. doi: 10.1177/0333102420937655 32594760

[39] Schytz HW Birk S Wienecke T Kruuse C Olesen J Ashina M. PACAP38 induces migraine-like attacks in patients with migraine without aura. Brain. (2009) 132:16–25. doi: 10.1093/brain/awn307, PMID: 19052139

[40] Ashina M. Migraine. N Engl J Med. (2020) 383:1866–76. doi: 10.1056/NEJMra1915327 33211930

[41] Retamales-Ortega R Vio CP Inestrosa NC. P2C-type ATPases and their regulation. Mol Neurobiol. (2016) 53:1343–54. doi: 10.1007/s12035-014-9076-z, PMID: 25631710

[42] Avalos Prado P Chassot AA Landra-Willm A Sandoz G. Regulation of two-pore-domain potassium TREK channels and their involvement in pain perception and migraine. Neurosci Lett. (2022) 773:136494. doi: 10.1016/j.neulet.2022.136494, PMID: 35114333

[43] Avalos Prado P Landra-Willm A Verkest C Ribera A Chassot AA Baron A. TREK channel activation suppresses migraine pain phenotype. iScience. (2021) 24:102961. doi: 10.1016/j.isci.2021.102961, PMID: 34458705 PMC8379698

[44] Royal P Avalos Prado P Wdziekonski B Sandoz G. Canaux potassiques à deux domaines P (K2P) et migraine. Biol Aujourdhui. (2019) 213:51–7. doi: 10.1051/jbio/2019020, PMID: 31274103

[45] Oka F Lee JH Yuzawa I Li M von Bornstaedt D Eikermann-Haerter K. CADASIL mutations sensitize the brain to ischemia via spreading depolarizations and abnormal extracellular potassium homeostasis. J Clin Invest. (2022) 132:e149759. doi: 10.1172/JCI149759, PMID: 35202003 PMC9012276

[46] Ekmekcioglu C Elmadfa I Meyer AL Moeslinger T. The role of dietary potassium in hypertension and diabetes. J Physiol Biochem. (2016) 72:93–106. doi: 10.1007/s13105-015-0449-1 26634368

[47] Gritter M Wouda RD Yeung SMH Wieers MLA Geurts F de Ridder MAJ. Effects of short-term potassium chloride supplementation in patients with CKD. J Am Soc Nephrol. (2022) 33:1779–89. doi: 10.1681/ASN.2022020147 35609996 PMC9529195

[48] Sun Y Byon CH Yang Y Bradley WE Dell'Italia LJ Sanders PW. Dietary potassium regulates vascular calcification and arterial stiffness. JCI Insight. (2017) 2:e94920. doi: 10.1172/jci.insight.94920 28978809 PMC5841863

[49] Ebrahim Amini A Bazzigaluppi P Aquilino MS Stefanovic B Carlen PL. Neocortical in vivo focal and spreading potassium responses and the influence of astrocytic gap junctional coupling. Neurobiol Dis. (2021) 147:105160. doi: 10.1016/j.nbd.2020.105160, PMID: 33152505

[50] Harrington MG Fonteh AN Cowan RP Perrine K Pogoda JM Biringer RG. Cerebrospinal fluid sodium increases in migraine. Headache. (2006) 46:1128–35. doi: 10.1111/j.1526-4610.2006.00445.x, PMID: 16866716

[51] Zhang J Mao Y Li Y Zhao K Xie Q Wang K. Association between migraine or severe headache and hypertension among US adults: a cross-sectional study. Nutr Metab Cardiovasc Dis. (2023) 33:350–8. doi: 10.1016/j.numecd.2022.11.014 36604265

[52] Zhang Y Parikh A Qian S. Migraine and stroke. Stroke Vasc Neurol. (2017) 2:160–7. doi: 10.1136/svn-2017-000077 28989805 PMC5628377

