# Supplementary material for: Association between dietary potassium intake and severe headache or migraine in US adults: a population-based analysis
Source: Front Nutr. 2023 Sep 15;10:1255468. doi: 10.3389/fnut.2023.1255468 (PMC10540813; doi:10.3389/fnut.2023.1255468)
Supplement: Supplementary file 1 [file Table_1.docx]

**Supplementary Table1. Association of covariates and migraine risk.**

| Variable | OR (95% CI) | *p*-Value |  | Variable | OR (96% CI) | *p*-Value |
| --- | --- | --- | --- | --- | --- | --- |
| Age(years) | 0.98 (0.97~0.98) | <0.001 |  | Physical activity, n (%) |  |  |
| Sex, n (%) |  |  |  | Sedentary | 1(reference) |  |
| Male | 1(reference) |  |  | Moderate | 0.85 (0.75~0.95) | 0.005 |
| Female | 2.16 (1.96~2.39) | <0.001 |  | Vigorous | 0.82 (0.73~0.92) | 0.001 |
| Race/ethnicity, n (%) |  |  |  | Hypertension, n (%) |  |  |
| Non-Hispanic white | 1(reference) |  |  | No | 1(reference) |  |
| Non-Hispanic black | 1.3 (1.14~1.47) | <0.001 |  | Yes | 0.96 (0.86~1.08) | 0.519 |
| Mexican American | 1.23 (1.09~1.39) | 0.001 |  | Diabetes, n (%) |  |  |
| Others | 1.38 (1.16~1.64) | <0.001 |  | No | 1(reference) |  |
| Education level (years), n (%) |  |  |  | Yes | 0.92 (0.78~1.08) | 0.292 |
| <High school | 1(reference) |  |  | Stroke，n (%) |  |  |
| High school | 1.13 (0.98~1.31) | 0.1 |  | No | 1(reference) |  |
| >High school | 0.88 (0.76~1.01) | 0.076 |  | Yes | 1.18 (0.91~1.53) | 0.215 |
| Marital status, n (%) |  |  |  | Coronary heart disease, n (%) |  |  |
| Married or living with a partner | 1(reference) |  |  | No | 1(reference) |  |
| Living alone | 1.11 (1~1.22) | 0.041 |  | Yes | 0.64 (0.5~0.83) | 0.001 |
| Family income, n (%) |  |  |  | Body mass index(kg/m2) | 1.02 (1.01~1.03) | <0.001 |
| Low | 1(reference) |  |  | Dietary intake |  |  |
| Medium | 0.73 (0.66~0.82) | <0.001 |  | Energy intake(kcal/d) | 1 (1~1) | 0.514 |
| High | 0.53 (0.47~0.6) | <0.001 |  | Protein intake(g/d) | 1 (1~1) | 0.011 |
| Smoking status, n (%) |  |  |  | Carbohydrate intake(g/d) | 1 (1~1) | 0.052 |
| Never | 1(reference) |  |  | Fat intake(g/d) | 1 (1~1) | 0.6 |
| Current | 1.29 (1.15~1.45) | <0.001 |  | potassium intake(mg/d) | 1 (1~1) | <0.001 |
| Former | 0.69 (0.61~0.78) | <0.001 |  | Sodium intake(mg/d) | 1 (1~1) | 0.058 |
| C-Reactive Protein(mg/dl) | 1.04 (1~1.09) | 0.066 |  | Magnesium intake(mg/d) | 1 (1~1) | <0.001 |
| Heart failure, n(%) |  |  |  | Angina, n(%) |  |  |
| No | 1(reference) |  |  | No | 1(reference) |  |
| Yes | 0.87 (0.66~1.17) | 0.362 |  | Yes | 1.17 (0.93~1.49) | 0.181 |
| Heart attack |  |  |  |  |  |  |
| No | 1(reference) |  |  |  |  |  |
| Yes | 0.84 (0.66~1.07) | 0.149 |  |  |  |  |

Univariate logistic regression was applied to analyse the relationship between each covariate and migraine.
