# Supplementary material for: Association between dietary potassium intake and severe headache or migraine in US adults: a population-based analysis
Source: Front Nutr. 2023 Sep 15;10:1255468. doi: 10.3389/fnut.2023.1255468 (PMC10540813; doi:10.3389/fnut.2023.1255468)
Supplement: Supplementary file 2 [file Table_2.doc]

**Supplementary Table 2. Dietary potassium among US adults in NHANES 1999-2004.**

| Sex | Total (%) | Threshold (mg/day) | | *P* value | potassium (mg/day) | | *P* value |
| --- | --- | --- | --- | --- | --- | --- | --- |
|  |  | <1439.3 | ≥1439.3 |  | <3500 | ≥3500 |  |
| Male | 5163（50.4%） | 537（34.2%） | 4626（53.3%） | <0.001 | 3565 (44.6%) | 1598 (70.8%) | <0.001 |
| Female | 5091(49.6%) | 1032（65.8%） | 4059（46.7%） |  | 4431 (55.4%) | 660 (29.2%) |  |
